# The Book World of Medicine and Science

**Published:** 1907-12-07

**Authors:** 


### The Book World of Medicine and Science.

Diseases of Women—A Clinical Guide to their Diagnosis and Treatment. By George Ernest Herman, M.B.Lond., F.R.C.P., Consulting Obstetric Physician to the London Hospital. (London. Cassell and Co., Ltd. 3rd edition. pp. 900, with 265 illustrations. Price 12s. 6d.)

The fact that this work has reached its third edition is clear proof of its popularity. It is essentially a clinical text-book which will appeal more to the practitioner than to the student preparing for examinations. It cannot claim to be complete from the pathological point of view, and the author admits in his preface that "this is not a book on histology." This work, when it first appeared, was welcomed as a great advance, and did much to place clinical gynæcology on a sound basis and to dispel many of the fallacies of the older writers. As a clinical work it is of the greatest value, and the practitioner will not turn to it in vain for guidance in diagnosis and treatment. The author assumes that his readers have been well grounded in aseptic surgery, and no doubt he is justified in this assumption, but surgical technique is very easily forgotten, and vaginal operations are apt to be performed after exceedingly imperfect preparation and cleansing. It is quite certain that douching with 1 in 2,000 perchloride of mercury solution is not a sufficient method of preparing the vagina for an operation. It cannot but happen that we are at variance with some of the opinions advanced, but taken as a whole the author expresses clearly the gist of modern teaching, and we cordially recommend the book to men already in practice.

Manual of Midwifery By W. E. Fothergill, M.A., B.Sc., M.D., Lecturer in Obstetrics, Victoria University, Assistant Gynæcologist to the Northern Hospital for Women and Children and St. Mary's Hospital for Women and Children, Manchester. (William Green and Sons, Edinburgh and London. 4th edition, 1907, pp. 488, with 103 illustrations and coloured plate. Price 4s. 6d. net.)

This is a most excellent text-book of midwifery, and can be cordially recommended to students. It is characterised by sound common sense and absence of unsustained theory throughout—very excellent features in a text-book of midwifery. It has been thoroughly revised, parts re-written, and new illustrations added. Among other chapters that on the early development of the human ovum, and its method of embedding in the uterine wall has been completely brought up to date, and is well written. The references to Hart's work on the mechanism of labour and Milne Murray's on the forceps cannot fail to be appreciated. Indeed, the mechanism of labour, as described by the author, has always struck us as one of the prominent features of the book, and we think he might well have completed the demolition of the old lever theory by pointing out how utterly it fails in cases of occipito-posterior presentations. The author is not quite fair in his criticism of the treatment of placenta prævia by de Ribes' bag, and we are sure that most authorities will not agree with him. With regard to accidental hæmorrhage he does not do justice to the plugging treatment now so warmly advocated by the Dublin school, and we cannot but feel that serious cases of accidental hæmorrhage will continue to end fatally, if treated by the old method of rupturing the membranes and rapid delivery. Apart from these few points for criticism the author presents clearly the modern teaching of the subject; his complete but not unwieldy treatise will probably continue to enjoy its well-deserved popularity.

Gout: Its Pathology, Forms, Diagnosis, and Treatment. By Arthur P. Luff, M.D., B.Sc., F.R.C.P. Third Edition. (London: Cassell and Company, Ltd., 1907. Pp. xii. and 290. Price 10s. 6d. net.)

The reputation of Dr. Luff's manual as an authoritative work on the subject with which it deals is already well-established, and in the new edition due note has been taken both of modern developments of doctrine and of recent improvements in treatment. Hence the book is one to which appeal may be made with confidence. Dr. Luff's chemical researches have, without question, appreciably advanced our knowledge of the metabolic changes which attend the gouty state, though the ultimate secret of the disease has not yet been penetrated. In the present work the influence of these researches on medicinal and dietetic treatment is well displayed, and here are to be found many contributions of distinct therapeutic value. Indeed, the subject of treatment is a conspicuous feature in the book, and is presented with the authority which arises from a carefully-studied personal experience. A new feature of the present edition is the inclusion of a special chapter on the differential diagnosis of the various chronic diseases of the joints. In this an attempt is made to provide a ready means of distinguishing between gout, rheumatism, rheumatoid, and other forms of arthritis. Possibly, until an etiological method is devised, this ambition will never be fully successful. But Dr. Luff's chapter is certainly a useful one even though it cannot be considered an absolutely satisfactory guide.

An Index of Treatment. By Various Authors. Edited by Robert Hutchison, M.D., F.R.C.P., and H. Stansfield Collier, F.R.C.S., Bristol. John Wright and Co. 21s. net.

There are various text-books of treatment to which the practitioner and the senior student can refer. None of them will serve instead of the experience to be gained by personal observation in hospital and at the bedside, but most of them have good points which make them valuable reference books for the practitioner's library. Dr. Hutchison and Mr. Collier's book will in a large measure supply the need which most medical men in general practice feel for a handy reference volume on the essentials of treatment. It combines surgical and medical treatment, it contains articles which are short essays in themselves, and it gives as full and as complete information as one can desire to obtain within the limits of a crown-octavo volume. The list of contributors is one that inspires respect and confidence; on each side the writers are authorities on their respective subjects, and the editing of the work has been so ably done that, while each article retains the stamp of individual authorship, there is yet a uniformity of arrangement which prevents the book from being a mere collection of stray notes on treatment. The information given is wholly sound, and new methods yet on their trial are only briefly referred to. At the same time, in a few of the articles old lines of treatment which appear to be out of date was given. The insistence on "methods of flagellation" (as we believe Professor Allbutt called them) in the treatment of opium poisoning is an anachronism in so up-to-date a volume. It would be invidious to single out articles for special mention. All are excellent, a few outstandingly so, and the book is one which we can cordially commend to the attention of practitioners and senior students who wish to possess a thoroughly sound, ably written, and informative work on the essentials of medical and surgical treatment.

